# Pediatric ovarian torsion: a diagnostic challenge

**DOI:** 10.1590/0100-3984.2016.0227

**Published:** 2018

**Authors:** Chiyyarath Gopalan Muralidharan, Shyam Krishna, Tony Jose

**Affiliations:** 1 Command Hospital (SC) Pune, Maharashtra, India; 2 Armed Forces Medical College, Pune, Maharashtra, India

**Figure 1:**
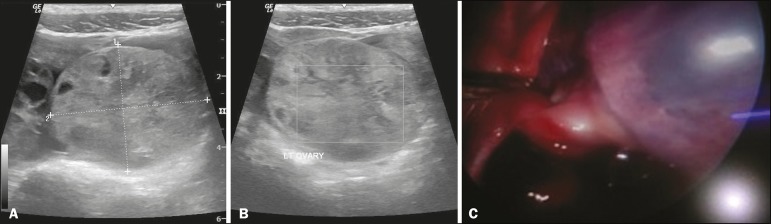
**A:** Ultrasound showing enlarged echogenic left ovary. **B:** No vascularity seen in the left ovary on color Doppler. **C:** Laparoscopic appearance of the left ovary.

Dear Editor,

A 12-year-old female presented with a 6-h history of acute severe lower abdominal pain in the hypogastrium and left iliac fossa, together with episodes of vomiting. Physical examination revealed a soft abdomen with severe tenderness in the hypogastrium and left iliac fossa. Blood test results were normal. Ultrasound revealed and an enlarged (~ 52 mL) echogenic left ovary (Figure 1A) with free fluid surrounding the ovary and in the pelvic cavity. No cystic or solid lesion was identified within the enlarged ovary. Color Doppler (Figure 1B) revealed no vascularity in the enlarged ovary. The right ovary was normal in size (~ 9 mL). The patient underwent urgent laparoscopy, which revealed an enlarged, congested left ovary (Figure 1C), and left oophorectomy was performed. Histopathology confirmed the diagnosis of ovarian torsion.

Ovarian torsion is the fifth leading gynecological condition requiring emergency surgery^(1)^. Delayed diagnosis can lead to unsalvageable ovaries and complications like peritonitis. The dilemma in the diagnosis is due to the relative rarity of the condition (incidence, ~ 2-3%), especially in children, as well as to the nonspecificity of the symptoms and the other varied etiologies that take precedence over ovarian torsion in children^(2)^.

Ovarian torsion is defined as the twisting of the ovary on its pedicle, leading to vascular obstruction. Pathophysiologically, the venous outflow is obstructed, resulting in congestion and hemorrhagic infarcts, which in turn result in arterial impairment^(3)^. It is more common in women of reproductive age, including pregnant women, probably due to the higher incidence of physiological and pathological masses in that age group^(4,5)^. It is relatively rare in the pediatric population. The symptoms are nonspecific and, due to the rarity of this condition in pediatric patients, etiologies such as appendicitis, diverticulitis, and renal colic are more likely explanations for the clinical symptoms than is ovarian torsion. Ultrasound is the first-line imaging modality in any case of acute abdomen.

One study showed that ultrasound has a positive predictive value of 87.5% and a specificity of 93.3% for the diagnosis of ovarian masses, as well as having other advantages such as low cost, easy accessibility, and no radiation^(6)^. On gray-scale imaging, the affected ovary appears enlarged, increasing to up to 28 times its original size^(7)^. The diagnostic criteria for enlarged ovaries include an ovarian diameter of > 4 cm or volume > 20 mL in women of reproductive age and > 10 mL in postmenopausal women^(8,9)^. Cystic or solid masses can also be identified on ultrasound. Cysts can show wall thickening. Free fluid can be seen in the pelvic cavity. The twisted vascular pedicle is typically seen as an echogenic round or beaked mass with multiple concentric, hypoechoic, target-like stripes. It can also appear as an ellipsoid or tubular mass with internal heterogeneous echoes, depending on the plane of orientation. Although color Doppler typically shows the absence of arterial flow, the presence of arterial flow does not rule out the possibility of torsion, because the arteries are affected at a later stage and there can be arterial supply from the uterine arteries as well. The twisted vascular pedicle can give rise to the whirlpool sign on color Doppler.
